# Supplementary material for: Effect of Grazing Management on Predator Soil Mite Communities (Acari: Mesotigmata) in Some Subalpine Grasslands from the Făgăraş Mountains—Romania
Source: Insects. 2023 Jul 12;14(7):626. doi: 10.3390/insects14070626 (PMC10380866; doi:10.3390/insects14070626)
Supplement: Supplementary file 1 [file insects-14-00626-s001.zip › insects-2437731-supplementary.pdf]

Effect of Grazing Management on Predator Soil Mite Communities (Acari: Mesotigmata) in Some Subalpine Grasslands from the Făgăraş Mountains—Romania

Minodora Manu [1,*,†], Raluca Ioana Băncilă [2,3] and Marilena Onete [1,*,†]

[1] Department of Taxonomy, Ecology and Nature Conservation, Institute of Biology Bucharest, Romanian Academy, Street Splaiul Independenţei, 060031 Bucharest, Romania
[2] Faculty of Natural and Agricultural Sciences, University Ovidius Constanţa, 900470 Constanţa, Romania; raluca.ioana.bancila@univ-ovidius.ro
[3] Department of Biospeleology and Soil Edaphobiology, "Emil Racoviţă" Institute of Speleology, Romanian Academy, 050711 Bucharest, Romania
* Correspondence: minodora.manu@ibiol.ro (M.M.); marilena.onete@ibiol.ro (M.O.); Tel.: +40-212-219-202 (M.M. & M.O.); Fax: +40-212-219-071 (M.M. & M.O.)
† These authors contributed equally to this work.

Table S1. Results of analysis of variance (ANOVA) in the generalized linear mixed effects models (GLMM) procedure testing the effect of grassland type and environmental variables on mite diversity. Statistically significant results are in bold. Df - degree of freedom; χ2 - chi-square test; P - significance level; GT - grassland type.

		Abundance		Species richness		Shannon-Wiener diversity index	
	Df	χ2	P	χ2	P	χ2	P
GT	1	0.289	0.591	0.095	0.757	0.001	0.978
Tsoil	1	0.895	0.344	0.012	0.912	0.908	0.340
Usoil	1	0.006	0.940	0.808	0.368	0.084	0.772
RP	1	0.023	0.878	0.102	0.749	0.675	0.411
pH	1	0.359	0.549	0.089	0.765	0.008	0.930
CE	1	1.114	0.291	5.473	0.019	3.635	0.057
VegCov	1	17.401	<0.001	7.243	0.007	2.370	0.124

Table S2: Numerical abundance and some population parameters of soil mites' communities from ungrazed and intensive grazed grasslands from Făgăraş Mountains, in 2018, from Romania.

No crt	Species	Short name	Sun	Cun	Vun	Gun	Total ungrazed	Vg	Sg	Cg	Gg	Total intensive grazed	Total grazed+ungrazed
1	*Alliphis halleri*	Alli hall	0	0	0	0	0	0	64	0	0	64	64
2	*Amblyseius sp.*	Ambl sp	0	2	0	0	2	0	0	0	0	0	2
3	*Ameroseius sp.*	Amer sp	1	0	0	0	1	0	0	0	0	0	1
4	*Arctoseius cetratus*	Arct cetr	7	0	0	0	7	0	13	0	0	13	20
5	*Arctoseius eremitus*	Arct erem	0	0	0	1	1	0	0	0	0	0	1
6	*Arctoseius resinae*	Arct resi	0	0	0	0	0	1	0	0	0	1	1
7	*Asca bicornis*	Asca bico	0	1	0	0	1	0	0	0	0	0	1
8	*Gamasellodes insignis*	Gama insi	0	0	0	0	0	0	1	0	0	1	1
9	*Geolaelaps aculeifer*	Geol acul	0	0	0	2	2	0	0	0	0	0	2
10	*Geolaelaps nolli*	Geol nolli	3	0	0	0	3	0	0	0	0	0	3
11	*Geolaelaps praesternalis*	Geol prae	3	7	5	4	19	0	9	5	2	16	35
12	*Iphidonopsis sculptus*	Iphi scul	0	0	0	1	1	0	0	0	0	0	1
13	*Iphidozercon gibbus*	Iphi gibb	0	0	0	0	0	0	2	0	0	2	2
14	*Leiodinychus orbicularis*	Leio orbi	0	0	0	0	0	0	0	0	1	1	1
15	*Leptogamaus tectegynellus*	Lept tect	1	0	0	0	1	0	0	0	0	0	1
16	*Lysigamasus sp.*	Lysi sp	0	2	0	0	2	0	0	1	0	1	3
17	*Onchodellus alpinus*	Onch alpi	2	0	0	0	2	0	1	0	0	1	3
18	*Oodinychus ovalis*	Oodi oval	1	0	0	0	1	0	0	0	0	0	1
19	*Parasitus sp.*	Para sp	1	1	0	0	2	0	0	0	0	0	2
20	*Pergamasus crassipes*	Perg cras	0	1	0	0	1	0	0	0	0	0	1
21	*Pergamasus quisquiliarum*	Perq quis	1	0	0	0	1	0	0	0	0	0	1
22	*Punctodendrolaelaps sp.*	Punc sp	5	0	0	0	5	0	0	0	0	0	5
23	*Trachytes aegrota*	Trac aegr	0	0	0	0	0	0	0	0	1	1	1
24	*Urodiaspis sp.*	Urod sp	0	0	0	1	1	0	0	0	0	0	1
25	*Veigaia exigua*	Veig exig	0	0	0	0	0	2	0	0	0	2	2
26	*Veigaia nemorensis*	Veig nemo	1	0	1	0	2	0	4	0	0	4	6
27	*Vulgarogamasus kraepelini*	Vulg krae	0	2	0	0	2	0	0	0	0	0	2

28	*Zercon carpathicus*	Zerc carp	0	7	0	2	9	0	6	0	1	7	16
29	*Zercon fageticola*	Zerc fage	0	0	0	0	0	1	0	0	0	1	1
30	*Zercon sp.*	Zerc sp	3	0	0	0	3	0	0	0	0	0	3
	No. of species		12	8	2	6	22	8	2	3	4	14	30
	No. of individuals		29	23	6	11	69	4	100	6	5	115	184
	Dominance_D		0.132	0.214	0.722	0.223	0.120	0.440	0.722	0.375	0.280	0.348	0.181
	Simpson_1-D		0.868	0.786	0.278	0.777	0.880	0.560	0.278	0.625	0.720	0.652	0.820
	Shannon_H		2.231	1.770	0.451	1.642	2.587	1.235	0.451	1.040	1.332	1.564	2.306
	Evenness_e^H/S		0.776	0.734	0.785	0.861	0.604	0.430	0.785	0.943	0.947	0.341	0.335
	Equitability_J		0.898	0.851	0.650	0.916	0.837	0.594	0.650	0.946	0.961	0.593	0.678
